# Nanoscopic and Photonic Ultrastructural Characterization of Two Distinct Insulin Amyloid States

**DOI:** 10.3390/ijms13021461

**Published:** 2012-02-01

**Authors:** Katarzyna Maria Psonka-Antonczyk, Julien Duboisset, Bjørn Torger Stokke, Tamotsu Zako, Takahiro Kobayashi, Mizuo Maeda, Sofie Nyström, Jeff Mason, Per Hammarström, K. Peter R. Nilsson, Mikael Lindgren

**Affiliations:** 1 Department of Physics, Norwegian University of Science and Technology,7491 Trondheim, Norway; E-Mails: katarzyna.psonka-antonczyk@ntnu.no (K.M.P.-A.); julien.duboisset@fresnel.fr (J.D.); bjorn.stokke@ntnu.no (B.T.S.); 2 Bioengineering laboratory, RIKEN Institute, 2-1 Hirosawa, Wako-shi, Saitama 351 0198, Japan; E-Mails: zako@riken.jp (T.Z.); tk65249@gmail.com (T.K.); mizuo@riken.jp (M.M.); 3 Department of Physics, Chemistry and Biology, Linköping University, Linköping 581 83, Sweden; E-Mails: sofny@ifm.liu.se (S.N.); jefma@ifm.liu.se (J.M.); perha@ifm.liu.se (P.H.); petni@ifm.liu.se (K.P.R.N.)

**Keywords:** amyloid proteins, oligomeric amyloid state, pre-fibrillar intermediate state, oligothiophene fluorescence stains, fluorescence assay, TIRFM-AFM

## Abstract

Two different conformational isoforms or amyloid strains of insulin with different cytotoxic capacity have been described previously. Herein these filamentous and fibrillar amyloid states of insulin were investigated using biophysical and spectroscopic techniques in combination with luminescent conjugated oligothiophenes (LCO). This new class of fluorescent probes has a well defined molecular structure with a distinct number of thiophene units that can adopt different dihedral angles depending on its binding site to an amyloid structure. Based on data from surface charge, hydrophobicity, fluorescence spectroscopy and imaging, along with atomic force microscopy (AFM), we deduce the ultrastructure and fluorescent properties of LCO stained insulin fibrils and filaments. Combined total internal reflection fluorescence microscopy (TIRFM) and AFM revealed rigid linear fibrous assemblies of fibrils whereas filaments showed a short curvilinear morphology which assemble into cloudy deposits. All studied LCOs bound to the filaments afforded more blue-shifted excitation and emission spectra in contrast to those corresponding to the fibril indicating a different LCO binding site, which was also supported by less efficient hydrophobic probe binding. Taken together, the multi-tool approach used here indicates the power of ultrastructure identification applying AFM together with LCO fluorescence interrogation, including TIRFM, to resolve structural differences between amyloid states.

## 1. Introduction

Amyloid fibrils form when certain proteins and peptides misfold and self-associate in an abnormal manner [1]. Even though the amyloidogenic precursor proteins possess completely different native protein structures and different primary sequences, the aberrant self-assembly leads to final fibrillar structures sharing a number of structural properties, such as the pronounced cross-β-sheet secondary structure and unbranched fibril morphology [2]. Extracellular deposits of amyloid fibrils are associated with a number of diseases including Alzheimer’s disease (AD), systemic amyloidoses, maturity onset diabetes, and prion-related transmissible spongiform encephalopathies [3,4]. Results from several groups have demonstrated that the conformational conversion from a native protein towards a fibrillar state is a phenomenon common to many proteins and peptides, even for proteins of various organisms, which are not involved in amyloid disease states, e.g., the *N*-terminal domain of HypF from *Escherichia coli* (Hyp-F-N) [5], and human tissue factor [6]. Findings such as these have lead to the hypothesis that amyloid fibril formation is a generic property of the poly-peptide backbone [7] not limited to a few specific sequences. Amyloid fibrils are also known to be cytotoxic [8–10] although the details behind the cell death mechanisms remain unclear. Several studies suggest that cell death might occur through membrane damage induced by amyloid and/or signal pathways via several surface receptors [11,12].

The fibrillar structures and related protein states have been studied using established state-of-the-art protein characterization techniques such as small angle X-ray diffraction [13] and NMR [14] throughout the past 20 years, however, detailed experimental 3D structures of the prefibrillar and fibrillar states are still sparse [13–15]. Moreover, the generic fibrillar structures have also the ability to bind small molecules such as the widely used amyloid ligands thioflavin T (ThT) [16] and Congo red [17], with concomitant alterations of their optical properties in terms of e.g., fluorescence quantum efficiency and their influence of polarized light rendering their appearance birefringent. Using other small fluorescent molecules such as 4-(dicyanovinyl)julolidine (DCVJ) and 8-anilino-1-naphthalenesulfonic acid (ANS) that have high affinity to hydrophobic patches [18–20], along with fluorescent labelling of certain mutated proteins, supported determination of the kinetics and size distribution evolution during the fibrillation processes of several protein systems [18,21,22]. Luminescent conjugated poly- or oligothiophenes (LCPs and LCOs) have been developed over the past few years for studies of protein aggregates [23]. In contrast to the traditional fluorescent probes mentioned above, LCPs contain a twistable conjugated polymeric backbone usually based on repetitive thiophene units, whose conformational state affect their spectroscopic properties [24–26]. Binding to protein aggregates constrains the rotational freedom of the thiophene backbone, altering their spectral properties in a conformation-sensitive manner. Thus, an optical ‘fingerprint’ is obtained and this property has been used to discriminate prion protein aggregates associated with different prion strains [27,28], conformational heterogeneities in Amyloid-β amyloid plaques in Alzheimer disease mouse models [29], and morphologically different amyloid deposits in systemic amyloidoses [30]. LCPs and LCOs have also proven useful for detection of disease associated protein aggregates that go undetected by ThT and Congo red [27,28]. In addition, the LCPs have high multiphoton excitation capability [31], allowing *in vivo* studies of animal disease models [32].

It was previously shown that in the presence of a reducing agent, tris(2-carboxyethyl)phosphine, bovine insulin forms flexible filamentous protofibrillar amyloid assemblies which differed morphologically from intact insulin fibrillar amyloid [33]. Intriguingly, the cytotoxicity of the insulin filaments was remarkably lower than that of the insulin fibrils. This finding supports the idea that cell toxicity of amyloids correlates with their morphology, which in turn is dependent on the surface structure. Insulin filaments and fibrils can be a good model system for toxicity studies since they are formed from the same polypeptide. Thus, more detailed studies on these insulin amyloid structures are essential to elucidate both their differences in terms of morphology and physical properties as well as to understand how these relate to biological activity, e.g., in terms of toxicity.

**Figure 1:**
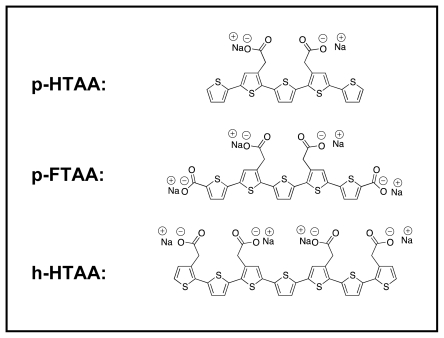
Chemical structures of LCO/LCP used in presented study: *p*-HTAA, *p*-FTAA, *h*-HTAA.

Here, the insulin fibrils and filaments were further studied using a series of three different LCOs [32,34] (Figure 1) with well-defined chain lengths, giving spectroscopically better resolved results than the commonly used LCPs [29–31,45]. The LCO probes used here have carboxylic groups attached to certain thiophene moieties. At pH values around the production of the amyloid morphologies (approx. pH 2) the side groups are neutral, whereas near physiological conditions at pH 7.5 (as in one of the assays used here) the acidic carboxylic groups are deprotonated, making the side-groups negatively charged. Change of side group charge depending on the surrounding pH can influence the binding properties between the protein and LCO. As is well known, salt bridges may form as a result of two non-covalent interactions: ionic interactions and hydrogen bonding. Such bridges usually arise from the anionic carboxylate (RCOO^−^) of either aspartic acid or glutamic acid and the cationic ammonium (RNH_3_^+^) from lysine or the guanidinium (RNHC(NH_2_)_2_^+^) of arginine [35]. Although each such interaction is fairly weak in water, they can add up to stabilize a protein conformation [36], or in the case of interactions between a relatively small molecule as an LCO, hydrogen bonds between RNH_3_^+^ and RCOO^−^ might serve as a stabilizer of an LCO at a binding side of an amyloidic structure. We examine the surface hydrophobicity and charge of the amyloids, and its impact on LCO binding. Atomic force microscopy (AFM) and fluorescence imaging spectroscopy was used in parallel to resolve their detailed structural properties with focus to gain basic insights into the amyloid specificity and the corresponding optical properties of the LCOs.

## 2. Results and Discussion

### 2.1. Preparation of Amyloidic Fibrillar and Filamentous Variants of Insulin

The fibrillar morphology of insulin amyloid was obtained using a standard procedure applying acidic conditions, similar to the technique used for producing insulin fibrils and its oligomeric precursors for X-ray studies [13]. The filaments on the other hand were prepared in presence of a reducing agent that breaks up intra-molecular S–S bonds within the A-chain or the intermolecular S–S bonds between the A- and B-chain of insulin prior to the onset of filament growth (see experimental section for more details). Thus, the identical sequences of amino acid residues were used in the two different preparation procedures, but as shown previously [33], bovine insulin can form two different morphologically amyloid states referred to as fibrils and filaments, respectively. The fibrillar and filamentous states display significantly different cytotoxicity [33].

Testing the toxicity and other biological actions of the two amyloid systems requires the use of buffer systems at pH higher than the one optimal for amyloid production. It was previously shown that significant ThT fluorescence can be observed for both insulin fibrils and filaments at neutral pH [33]. Using the very same approach, amyloidic states of insulin produced at pH 2 were checked for their stability in buffer usually used for molecular biology experiments, namely PBS (pH 7.4). Both fibrils and filaments showed similar ThT intensities even after 20 days incubation in PBS or MQ water, indicating that the two amyloids are stable over long time intervals in the neutral pH buffer and water (data not shown).

### 2.2. Characterization of Amyloid Surface Charge and Hydrophobicity

As bovine insulin contains 4 potentially cationic (Lysine; Arginine; 2× Histidine) and 4 potentially anionic residues (4× glutamic acid), it is likely that several of these remain exposed to the solvent phase after the fibril or filament state of the amyloid is formed. Ionic interactions can be particularly strong in a hydrophobic microenvironment. We investigated the surface charge potential of the amyloidic protein conformation and results of titration with the hydrophobic probe ANS for assessment of the nature of forces contributing to the LCO and LCP binding to the insulin fibril and filament variants.

In order to determine the surface charge, and thus net surplus of cationic or anionic unexposed residues, zeta-potential measurements were carried out. The zeta potential is affected by ionic strength and pH of the buffer, as well as thermodynamic properties such as temperature [37]. As dissolved LCO and LCP variants have their own p*K*_a_ values, they can act more or less as “free-ions” in the system, and these are likely to deteriorate the true surface potential value of the protein. Thus, the investigation was here limited to the pure fibril and filament aggregates at two different conditions: at pH 3.3 in acetic acid buffer (similar to the conditions for fibril and filament formation) and at pH 7.0 using a sodium phosphate buffer (resembling physiological conditions).

**Figure 2:**
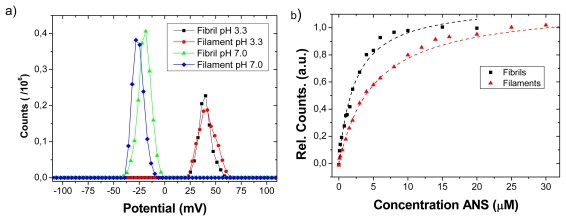
(**a**) Zeta potential measurements of insulin amyloidic fibrils and filaments with distributions determined at pH 7.0 (NaPi) and pH 3.3 (acetic acid); (**b**) Binding of ANS at pH 7.4 to insulin fibrils and filaments as judged from the onset of fluorescence.

Representative results of zeta potential measurements at the two different buffer systems are shown in Figure 2a. There are no significant differences on the observed positive zeta potential between fibrils and filaments at pH 3.3. The potential for both filaments and fibrils is negative at pH 7. Since fibrils and filaments were made from the same peptide sequence, the result suggests that cationic and anionic amino acid residues on the amyloid surface are similar when comparing fibrils and filaments. However we noted that the negative charge of filaments was slightly higher compared to fibrils at pH 7.0, indicating a subtle difference in surface charge.

A common approach to quantify hydrophobic patches on protein surfaces and interfaces is the fluorimetric assay based on hydrophobic probes such as ANS, see e.g., [19,20]. This procedure has previously been extensively used, for example for studies of ovalbumin at air-water interface [20], studies of the amyloid formation process of TTR [18]. The association of the ANS molecule to hydrophobic patches leads in an increase of fluorescence intensity by monitoring emission around 475 nm while exciting at 380 nm, as shown in Figure 2b. The dissociation constant of ANS for the fibrils and filaments was estimated to be 2.3 and 5.1 μM, respectively. Thus, the ANS assay indicates that the insulin amyloidic fibril surface is approximately twice as efficient to provide hydrophobic binding sites at pH 7.4. Taken together, the charge and hydrophobicity characterization suggests that there is a similar net charge surplus for the two types of amyloid structures, but of opposite charge at acid and neutral pH. The discrepancies between the two states were a slightly more negative charge for filaments at neutral pH and a stronger hydrophobicity for fibrils.

### 2.3. Fluorescence Spectral Fingerprint of Amyloid Molecular Targets

To get detailed information on the nature of the LCO binding to the insulin amyloid variants, a series of fluorescence spectroscopy and imaging experiments were carried out also including the neutral pH condition to find out whether the resulting details of the excitation and emission properties are different. Based on these findings we determined whether LCOs can recognize and even accommodate to the inner structures of the insulin amyloids.

**Figure 3:**
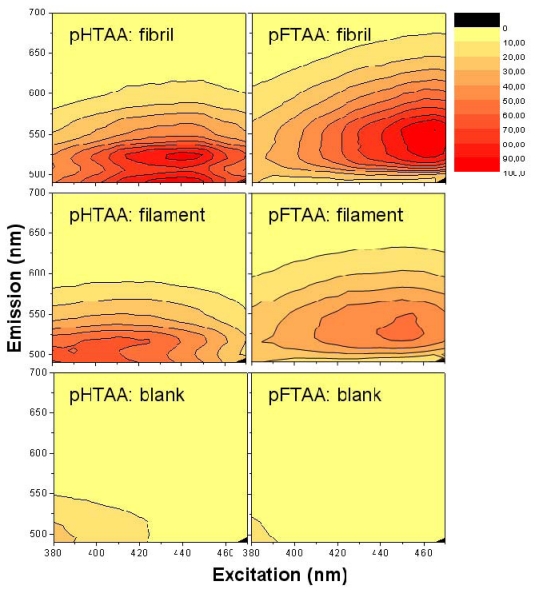
Excitation and emission spectra obtained at pH 2.0 directly after formation of fibrils and filaments staining with *p*-HTAA and *p*-FTAA. All 3D plots have the same intensity scale as shown in the bar to the right.

A convenient way to screen the fluorescent properties of the amyloid targets using LCPs and LCOs is to make a 3D fluorescence intensity plot, based on a 2D-scanning of excitation and emission. Such spectra are shown in Figure 3 using the LCOs *p*-HTAA and *p*-FTAA in the acidic pH buffer in which the fibrils and filaments are formed at pH 2.0. The spectra of the blank LCO samples at 0.2 μM are presented in the lower row, and the data taken with a 250 fold molar excess concentration of filament and fibrils (50 μM on a monomer basis) are collected as the upper and middle row, respectively. Also, the spectra are color-coded and normalized in such a way that each spectrum for each case has the same gain and absolute emission amplitude scale although all data points are normalized to the highest signal of all plots in one series (*i.e.*, all spectra in Figure 3) and this data point was set to 100.

For the two LCOs in acidic buffer there is almost no emission unless one goes down to excitation wavelengths well below 400 nm. On the other hand for the LCOs bound to the fibrils and filaments there is a large increase in quantum yield, especially for excitation around 430–450 nm. For particularly the fibrils, both *p*-HTAA and *p*-FTAA shows a dramatic shift in excitation upon binding to the amyloid and the smaller *p*-HTAA is blue shifted compared to *p*-FTAA. Comparing the spectra of fibrils and filaments, the latter is clearly more blue-shifted both in excitation and emission. Also, the spectra of the fibrils tend to show a more pronounced double-peak in emission whereas the fluorescence spectra of the filaments have less distinct features. From the appearance of the combined spectral excitation and emission patterns, it is evident that both amyloid states cause a spectral change, and thus binding likely occurs between the amyloid and luminescent probe at this low pH. The difference in both excitation and emission comparing the fibril and filament using either *p*-HTAA or *p*-FTAA suggest that the binding site is different in the two types of amyloids. Notably, for the cationic, amyloid specific, ThT fluorophore the intensity even in the presence of fibrils at acidic pH was 10 fold lower compared to the corresponding assay at pH 7.5, indicating charge repulsion (data not shown). The excitation and emission wavelengths of the LCOs, fibrils and filaments in other buffer systems at higher pH will be discussed further below.

### 2.4. Spectral Properties for LCO/Amyloid at Neutral pH

**Figure 4:**
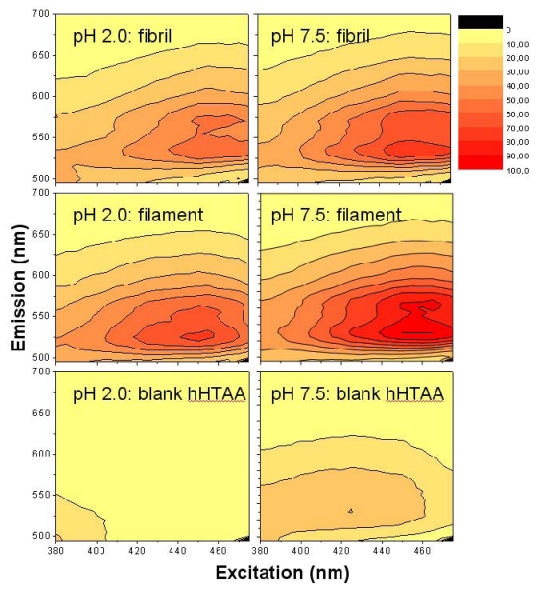
Excitation and emission spectra obtained at pH 2.0 and 7.5 for *h*-HTAA with insulin filaments and fibrils. Left column: recorded at pH 2.0 using acetic acid buffer. Right column: recorded at pH 7.5 using PBS buffer. Each row of plots is plotted using the same intensity scale as shown in the bar to the right.

As the surface charge measurements indicated slightly different net surface charge for the acid and neutral pH, it is relevant to examine the spectral fluorescence characteristics at the two conditions. The results of such measurements using the LCO *h*-HTAA with both the acetic acid buffer (pH 2.0) and PBS pH 7.5 buffers are shown in Figure 4. The data-points are normalized in a similar manner as for *p*-HTAA and *p*-FTAA shown in Figure 3.

The data show that *h*-HTAA (Figure 4) gives similar fluorescence responses as the smaller variants *p*-HTAA and *p*-FTAA (Figure 3), but the intensity for the filaments is here somewhat higher. There are also red-shifts of both the excitation and emission projections in comparison with the shorter LCOs *p*-HTAA and *p*-FTAA, as a result of the longer conjugation length over the thiophene framework giving a lower effective transition dipole moment and molecular band-gap. By dialyzing off small amounts of monomer remaining in the solution and changing the buffer to PBS pH 7.5, there were small changes in the spectral features, however the general features of the 3D plot remain similar. It is worth to note that the signal from the blank *h*-HTAA sample showed up with higher relative intensity at pH 7.5 than in the low pH case, but there were only minor changes in spectral characteristics comparing the result from low and neutral pH. Similar small differences were observed in the spectra at pH 7.5 for *p*-HTAA and *p*-FTAA (data not shown).

The very same samples used to obtain the 3D fluorescence plots in Figure 4 were also used to carry out a polarization anisotropy measurement by recording the difference in fluorescence with crossed and parallel polarizers for the excitation and emission. This corresponds to the “static” fluorescence anisotropy commonly used to deduce dynamic information of fluorophores introduced into proteins. An anisotropy of 0.27 was recorded for the fibrils in both the pH 2.0 and pH 7.5 systems. For the filaments the values obtained were similar 0.29 and 0.27, respectively. The value for the pure buffer solution could not be determined at low pH because of the poor signal, but for pH 7.5 an anisotropy of 0.15 was obtained. Thus, the increase in anisotropy from 0.15 to approximately 0.27 is a clear manifestation of a rather tight binding of the probe to the amyloid (see, e.g., ref [39]). Conclusively, the fibrils and filaments both showed LCO binding at both low and neutral pH, with a blue shift in emission for the filaments comparing with the fibril cases. Considering the lack of pH dependence on fluorescence anisotropy during binding, and as supported in the previous section on ANS binding, hydrophobicity is likely the dominating binding mechanism with a shallower and less defined binding pocket on the filaments compared to fibrils.

### 2.5. AFM of Insulin Fibril and Filament Morphology

The morphology of insulin fibrils and filaments was examined with TEM in a previous report [33]. In this study, more detailed structures of the insulin amyloids were examined using AFM (dried state) and AFM in combination with TIRFM (in buffer solution). For ultramicroscopy techniques, such as AFM and TIRFM, the purity of sample is one of the crucial parameters as well as full control over the process for stable attachment of the molecules of interest to the flat specimen surface. An immobilization strategy was established to make individual amyloids anchored to the glass or mica surface sufficiently strong to be able to remove free fluorescent dye, excess of salt, unbound aggregates and other unwanted particles (contamination) by rinsing with the solvent. These steps were essential to enhance signal to noise ratio of the detected signal and to avoid artifacts from salt deposits as well as from structures loosely bound/stacked to the substrate. A different strategy of anchoring was developed for each amyloid form (fibril and filament) and for each detection/visualization technique (AFM and correlated TIRFM/AFM) taking into account individual characteristics in terms of hydrophobicity and surface charge.

**Figure 5:**
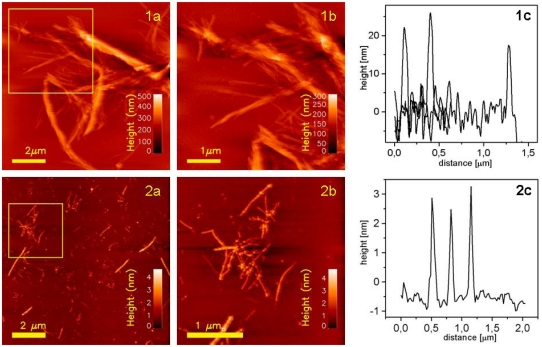
AFM topographs of insulin amyloids in dry state: (**1**) fibrils deposited onto gold-mica surface and (**2**) filaments anchored to the silane-mica. The yellow frames in 1a and 2a are zoomed in 1b and 2b, respectively. Plots 1c and 2c show representative height profiles from images 1b and 2b, respectively.

The morphology of the insulin amyloids was studied using AFM. Gold-modified surfaces were found to be applicable for attachment of fibrils for AFM imaging in both dry and hydrated states. However, since sufficiently stable binding of filaments to the gold-modified surfaces was not observed (data not shown), an alternative approach was needed to be able to prepare the filaments for ultramicroscopic imaging. Silane-modified substrate was explored as filament anchoring surface based on their net negative zeta potential. Dry samples of fibrils deposited onto Au-mica were imaged in tapping mode in air. Typical topographs are presented in Figure 5. Large aggregates of fibrils (see Figure 5:1a) reaching heights up to few hundreds of nanometers were detected and visualized. It was possible to collect zoomed topographs from smaller areas showing even more detailed structure within individual fibrils. Structural units aligned parallel along each other were visualized (see Figure 5:1b). Visualized fibrils seem to be rather rigid and compact structures. Filaments anchored to the silane-mica are showed on Figure 5:2a,b. Filamentous states present distinctive differences in sizes and structure, as compared to fibril states. They do not form complex ordered architectures. Instead, they exist as solitary, elongated curvilinear structures with average height approximately 3–4 nm. Compared to fibrils, they are more flexible as they show bent topographies. Large assemblies of filaments, if observable, were the result of random stacking of individual filaments rather than of building up ordered high level structures.

### 2.6. Fluorescence Spectral Imaging Using a Laser Scanning Microscope

Next step was to investigate the morphology of the fibrillar and filamentous amyloids from fluorescence microscopy. Using a confocal laser scanning microscope equipped with spectrally resolved PMT arrays, images with luminescence spectra in each pixel were obtained. By analyzing various regions of interest (ROIs), the spectral data as obtained in the previous sections could be related to the morphologic structures down to the approximate optical microscopy resolution limit.

**Figure 6:**
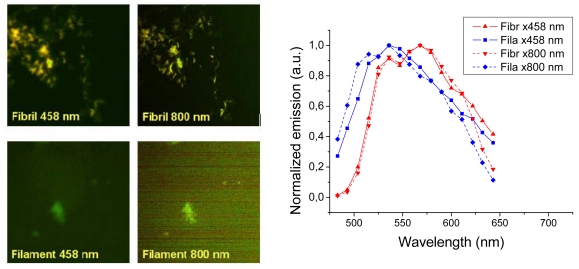
Images for *h*-HTAA associated to fibrillar and filamentous amyloid insulin for oneand two-photon excitation. Right graph: Emission spectra associated with a series of ROIs.

Representative images taken with a confocal laser scanning microscope are shown in Figure 6 (left panels). The fibril sample showed bright images both using one- (458 nm) and two-photon excitation (800 nm). The images show a distinct difference between the texture fibrillar and filamentous amyloids. Insulin fibrils appear as distinct thick fibrils some 0.2–0.5 μm in diameter, whereas the filaments showed up as unresolved “clouds”. Thus, just as the AFM showed that filament structures are much smaller than the fibrils, optical imaging has not the resolution required to see fine structure of the filaments. The spectral features of the two different amyloids were in agreement with the results measured in the fluorescence emission experiment (Figures 3 and 4), however, owing to the poorer resolution of the 32 channel meta detector of the laser scanning microscope, the emission spectra are broader and the features are not identical. In any case both one- and two-photon excitation give similar emission with a clear blue shift for the *h*-HTAA stained filaments compared to the fibrils (Figure 6, right). Experiments with *p*-FTAA and *p*-HTAA showed similar results (not shown). Conclusively, laser scanning microscopy did not reveal any further insight into the structural differences between the insulin fibrils and filaments bound to the different LCOs. As will be shown in the following section, much better resolution could be achieved using combined total internal reflection fluorescence (TIRF) imaging in combination with atomic force microscopy (AFM).

### 2.7. Combined AFM and TIRF Studies of Amyloid States

The fluorescence signal from LCOs that bound to the different insulin amyloid states was further investigated in correlated TIRFM/AFM experiments using the LCOs *h*-HTAA and *p*-FTAA. Hydrated samples were used for simultaneous observation of fluorescence signal and morphology of amyloid states at nanometer resolution. To achieve this, the TIRFM system was used in conjunction with AFM. Objective-based TIRF was used to collect fluorescence from the sample bottom whereas AFM mounted on the top of manipulation stage was used to record topographical maps of sample top. In this configuration, the sample was scanned in XY direction in TIRF mode to localize single amyloid aggregates based on its fluorescence and then sections were chosen for AFM to record their topographies.

**Figure 7:**
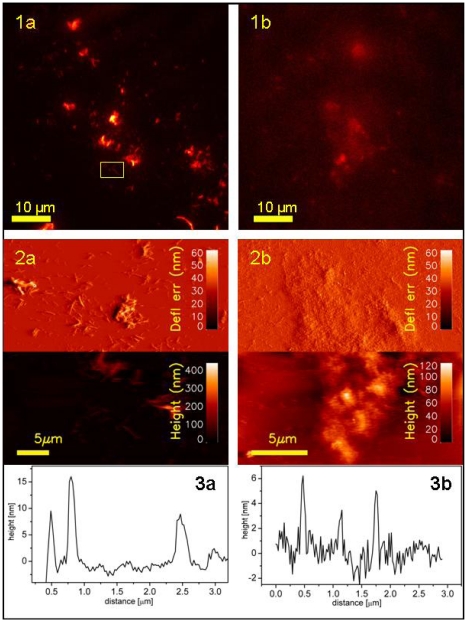
Characteristic images of single insulin amyloids–fibrils (panel a) and filaments (panel b): TIRFM fluorescence signal from *h*-HTAA-labeled fibrils (**1a**), and *p*-FTAA-labeled filaments (**1b**). Colocalized structural topography: contact mode AFM images (lower part, height; upper part, deflection error) for fibrils (**2a**) and filaments (**2b**). Plots (**3a**) and (**3b**) show height profiles from height images (**2a**) and (**2b**), respectively. Frame on image **1a** depicts an area that was chosen for further zooming (see Figure 8 for details).

Representative images of simultaneous fluorescence signal/topography of fibrils anchored to Au-glass are presented on Figure 7 panel (a). Large as well as small aggregates of fibrils are clearly visible. Due to the fact that the evanescence-wave-generated illumination has very narrow penetration depth (100–200 nm), the recorded signal originate only from the probe molecules that are attached to the parts of fibrils that are in the closest vicinity to the glass surface. Therefore, TIRFM records only a 2D projection of a part of individual fibril aggregates. The larger the aggregate the more intense is the collected fluorescence. Due to that fact, for fixed intensity range, a larger fibril is much better visible compared to small aggregates. Therefore, it is necessary to adjust dynamically intensity range depending on what size range of aggregates is of interest. Both TIRFM limitations, *i.e.*, recording signal only from the thin layer closest to the surface and the need of real-time readjusting intensity range to be able to observe full range of fibrils sizes, can be overcome when TIRFM is combined with colocalized recording of AFM topographs.

**Figure 8:**
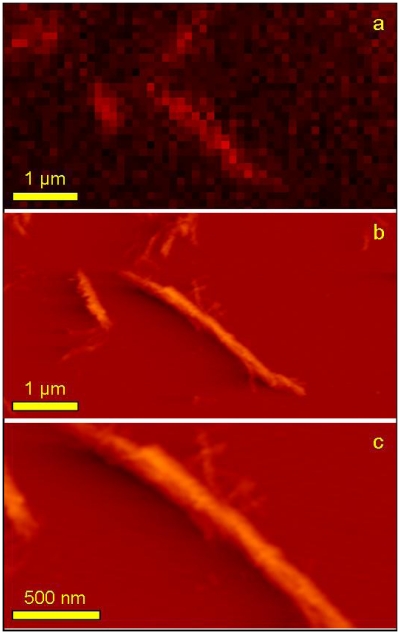
High magnification topographs of insulin amyloids. Zoomed area of TIRFM fluorescence (yellow frame in Figure 7:1a) showing individual small fibrils of insulin: (**a**) fluorescence signal; (**b**) AFM topograph of the same area as (**a**); (**c**) further zoomed area of image (**b**). Deflection error was chosen as representation of AFM topographs due to better contrast.

Using the TIRFM technique correlated with AFM imaging, it was possible to observe signal from probe molecules attached to the individual fibrils with high resolution and visualize the very same single fibril with the higher resolution of AFM (Figure 8). Figure 8a is a zoomed part of Figure 7:1a and its acquisition pixel size is 110 nm. The very same area but this time imaged with AFM is shown on Figure 8b and finally, an even more zoomed area showing a fragment of one long fiber is presented on Figure 8c. Both AFM topographs have acquisition pixel size equal to approximately 25 nm. It is clearly visible that the superior resolution of AFM topographs allows observing simultaneously the whole range of fibril sizes including the smallest structures not detectable with TIRFM. This is mostly due to the resolution limitation but also either due to the very low signal that is screened by much stronger signal from big aggregates or even the lack of the fluorescence signal (no/few probe molecules attached). Due to the limited sharpness of the AFM tip used for imaging (nominal value of apex curvature, *R* = 10 nm) it was not possible to reveal the periodicity of the imaged fibrils as it was reported in cryo-electron microscopy [44].

In parallel to the fibril samples, also filaments attached to sil-glass were studied using colocalized TIRFM/AFM imaging. Representative images of simultaneous fluorescence signal/topography of filaments are presented on Figure 7 panel b. Comparing the TIRFM image of filament on Figure 7 with its AFM analog, it is clearly visible that the fluorescence intensity depend on the level of filaments aggregation. Since the aggregates of filaments are the result of random stacking rather than directed build up of highly ordered structure, it is more difficult to detect such aggregation in fluorescence imaging. Filaments, in contrast to compact aggregates of fibrils, form more cloudy and spacious structures of loosely lying individual curvilinear particles.

## 3. Experimental Section

Insulin fibrils and filaments were prepared as described [33]. Bovine insulin (Sigma-Aldrich, ST Louis, MO, USA) was dissolved at a concentration of 20 mg/mL in 40 mM HCl (pH 1.5) and then immediately diluted in fibril formation buffer (20% acetic acid, 100 mM NaCl, pH 1.6) or filament buffer (20% acetic acid, 100 mM NaCl, pH 1.6, 20 mM tris(2-carboxyethyl)phosphine (TCEP)) at a protein concentration of 2 mg/mL (345 μM). Fibrillation was induced by heating the insulin solution at 70 °C for 13 h without agitation. In order to remove unassembled insulin monomer and exchange the buffer, samples were centrifuged at 15,000 rpm for 30 min. Fibril concentrations and conversion ratio from insulin monomer into insulin fibrils were determined beforehand by subtracting the insulin monomer concentration measured in supernatants after centrifugation at 15,000 rpm for 60 min of a part of the incubated insulin samples.

The stability of amyloid states was examined by storing insulin fibrils and filaments (at concentration 250 μM on a monomer basis) in buffer (PBS) or MQ water at room temperature. Aliquots (2 μL) were taken at desired time intervals from the stored sample mixture and added to a 100 μL solution containing 5 μM ThT in 10 mM phosphate, pH 7.4, 150 mM NaCl. Fluorescence measurements were carried out with a spectrofluorometer (FP-6500; Jasco, Tokyo, Japan) at 25 °C. The excitation wavelength was 450 nm and the emission was measured at 485 nm. The zeta potentials of insulin amyloids were determined using a Malvern Nano ZS90 system at 25 °C. Fifty μM of insulin fibrils and filaments samples in 20 mM acetic acid solution (pH 3.3) or 20 mM NaPi buffer (pH 7.0) were applied into the cell (DTS1060C-Cleatdisposable zeta cell). Zeta potentials were calculated from the electrophoretic mobility using a Smoluchowki relationship [37]. For hydrophobicity experiments, 10 μM of fibril and filaments were dissolved in 1.4 mL of 10 mM NaPi (pH 7.4) buffer and titrated in aliquots of 1.4 μL of 8-anilino-1-naphthalenesulfonic acid (ANS) up to approximately 20 μM.

The LCOs used here are depicted in Figure 1. The detail synthesis of the LCOs has been reported elsewhere [32,34]. The excitation and emission fluorescence maps of LCO stained fibrils and filaments were assessed at 50 μM of insulin (on a monomer basis) stained with 0.3 μM of LCO in 2 M acetic acid 0.5 M NaCl (pH 2) and in 50 mM phosphate, 100 mM NaCl (pH 7.5). Spectra were recorded using a Tecan Saphire2 fluorescence plate reader using a 3D setup with combined excitation (380–475 nm) and emission spectra (495–700 nm). Polarization anisotropy was measured by introducing polarizers for the excitation and emission light path. Excitation was here carried out at 470 nm (using a separate nano-LED) and recording the emission at 530 nm, using a 5 nm slit. The amyloid fibrils were visualized using a Zeiss 510-LSM equipped with a META spectral detector. Excitation was performed using a 458 nm HeNe laser and a Ti:Sapphire fs laser tuned to 800 nm used for two-photon excitation, for further details, see [31].

To immobilize fibrils and filaments for TIRF and AFM measurements, gold-modified surface (Au-glass, Au-mica) and silane-modified surface (sil-glass, sil-mica) was used as a substrate, respectively. Thin microscope coverslips (borosilicate coverslips, VWR International, West Chester, PA, USA) were cleaned in 1:1 v/v concentrated HCl/methanol for 30 min, then silanized using trimethoxysilylpropyldiethylenetriamine (1% in 1 mM acetic acid) for 30 min. After each step of modification, coverslips were rinsed with MQ-water and dried with a flow of compressed nitrogen. Freshly cleaved mica sheets (SPI Supplies, West Chester, PA, USA) were modified in the same way omitting the HCl/methanol cleaning. To produce gold-modified surfaces, coverslips cleaned with HCl/methanol were incubated for 15 min in 0.1% 3-mercaptopropyltrimethoxysilane in ethanol, followed by ethanol rinsing and dried using compressed nitrogen. Such SH-silane-modified glass and freshly cleaved mica sheets were used to sputter thin films (approximately 5–10 nm) of gold (Cressington 208HR, Cressington Scientific Instruments, Watford, UK). All prepared substrates were kept in dry environment until the amyloid immobilization experiments. All chemicals, if not stated otherwise, were purchased from Sigma Aldrich. Deionized water (resistivity 18.2 MΩ·cm, MilliQ-unit, Millipore, Bedfors, MA, USA), referred to as MQ-water, was used in all preparative steps. Mica and glass modification procedures were adjusted to our purposes based on previous protocol [38].

Insulin amyloids as dry samples were imaged with AFM. As substrate for AFM samples, mica disks were modified to optimize amyloids binding. Two different strategies of freshly cleaved mica modification were employed (see Results section for reasoning). Insulin fibrils and filaments were deposited onto Au-mica and silane-mica, respectively. A droplet of 10 μL of 50 μM amyloids (with 0.3 μM *h*-HTAA) in PBS was deposited onto a substrate, incubated for 5 min. and then rinsed with MQ-water. The latter step was crucial to avoid formation of solid deposits of salt onto the surface that could embed attached amyloids during the drying process and therefore introduce artifacts in the recorded AFM images. Rinsed substrates were dried in vacuum (~1–0.1 mPa). Samples were stored in ambient air in close container to limit contamination. Dried amyloids was imaged using AFM (Digital Instruments Multimode IIIa, Santa Barbara, CA, USA) operated in tapping mode under ambient conditions. Silicone nitride cantilevers PPP-NCH (Nanosensors, Neuchatel, Switzerland) were used (nominal resonance frequency: 204–497 N/m). Overlapping of trace and retrace signals were used as a prerequisite for adequate image acquisition.

For combined TIRFM/AFM imaging, a droplet of 10 μL of 50 μM fibril (filament) solution in PBS with 0.3 μM *h*-HTAA or *p*-FTAA was incubated for 10 min onto Au-glass (sil-glass). Then samples were rinsed with MQ-water to remove salt and free fluorescent probe molecules. Next, samples were dried in vacuum (~1–0.1 mPa). The samples were mounted in the non-sealed holder for the correlated TIRFM/AFM imaging. TIRFM imaging was performed using inverted optical microscope (Axio Observer A1D1, Zeiss, Jena, Germany) coupled with objective-based laser TIRF-system (100 mW multiline argon-ion laser, Zeiss, Jena, Germany) and EMCCD camera (iXon DU-897i, Andor Technology, Belfast, Northern Ireland). For TIRFM imaging, sealed samples were mounted onto XY manipulation stage and illuminated with 488 nm laser using a 525/50 nm band pass filter on the emission side. Images were collected using 100× oil immersion objective (total optics magnification was 160×) and EMCCD camera with EM (Electron Multiplying) Gain adjusted to optimize detected fluorescence signal. For coupled TIRFM/AFM study, TIRFM system was coupled with AFM setup (Bioscope II, Santa Barbara, CA, USA). Surface with amyloids was mounted onto XY scanner of AFM assembled on the inverted optical microscope. PBS was injected between AFM tip and surface as imaging and evanescence wave-generating medium. AFM imaging was performed in contact mode in liquid using Si_3_N_4_ cantilevers (Nanoprobe, NP-S, nominal spring constant *k* = 0.06 N/m). AFM was operated according to the manufacturer specifications. Overlapping of the trace and retrace path of scanning AFM tip was used as a perquisite for adequate image acquisition.

## 4. Conclusions

Insulin is a well-known and extensively studied model protein for amyloid fibril studies. Insulin is also reported to be present in some diabetic patients as subcutaneous deposits in iatrogenic insulin amyloidosis, following insulin injections. Heterogenous structures of amyloid deposits, popularly called amyloid strains, as well as smaller assembly forms with a plethora of morphological appearances are currently of vast interest in the amyloid field, as it is believed that amyloid structure and pathological activity appear correlated. LCPs are known to be a class of accurate spectroscopic fluorescent probe to disclose the nature of different amyloid deposits in both animal and human diseases, such as Alzheimer’s disease, prion diseases and systemic amyloidoses [1,3,7,8]. Here we aimed to investigate the preference of certain amyloid LCP probes, specifically the well-defined LCOs. As is well known one usually monitors the growth and presence of amyloid fibrils by exposing the *in vitro* formed fibrils to a fluorescent dye such as ThT, however ThT and similar stains do not show spectral differences upon binding, so it is hard to disclose any detailed information about the binding site. It was recently demonstrated that the two variants of insulin amyloids can be stained by PTAA (polythiophene acetic acid), giving different emission spectra and suggesting that the surface and inner structure are different [45]. As demonstrated here, with the LCOs it is possible to observe similar spectral shifts and spectral substructure. For a new way of mapping *in vitro* protein amyloid states with a selection of LCOs, we emphasized the use of spectral information to give a fingerprint of the amyloids. It means that we explore not only the emission of the probe as a function of some condition, but also its excitation spectrum. By scanning the emission for each excitation wavelength, we obtain more information; the 3D excitation-emission spectrum reveals their potential as providing a spectroscopic signature and classification of amyloid aggregate types [39]. As demonstrated here, the 3D fluorescence mapping (excitation, emission, intensity) is a useful tool for further studies using more advanced modes of detection, such as anisotropy and/or multiphoton mode of excitation. Comparing the LCOs: *p*-FTAA, *p*-HTAA, and *h*-HTAA, it is evident that these all bind strongly to the rigid fibril type of amyloid and with similar spectral changes in terms of a conspicuously red-shifted excitation spectrum and a well-defined substructure in both excitation and emission. For the curvilinear filaments the emission is blueshifted and with less resolved substructure. Thus, even for amyloids of very similar chemical origin the spectral signatures are different. By using a library of different LCO probes, we can identify and classify different types of amyloid conformations. Such work is currently in progress in our laboratories.

The surface charge of both the fibrils and filaments was the same at acidic pH. Slightly more negatively charged filaments compared to fibrils were observed at neutral pH. Even though the net surface charge shifted from negative to positive when going from pH 2.0 to pH 7.5, this had little effect the morphology as deduced from AFM and TIRF. Nevertheless the response to the spectral excitation and emission showed there are clear differences between the binding sites comparing the two kinds of amyloid states. The binding sites in both states are likely dominated by hydrophobic interactions due to the hydrophobic oligothiophene backbone, where charged moieties are placed differently around the hydrophobic grooves and these participate in stabilization of the binding. It is possible that the substructure showing up in both excitation and emission is due to unresolved vibrational structure to nearby hydrogen bonds between the LCO and positively charged groups.

AFM is one of the experimental methods frequently used in amyloid formation and topography studies [40–43]. In this study, AFM imaging providing visualization of amyloids at the nanometer level revealed the detailed information about the structure and architecture of individual fibrils and filaments. This technique showed ordered high-level structure within single stiff fibrils and random stacking of flexible filaments. TIRFM can be used as a good alternative to EM/TEM methods that are currently widely accepted as techniques for amyloid characterization. Having a stable and selective fluorescence probe that can be used for amyloid labelling, TIRFM imaging offers few advantages when compared to the mentioned electron microscopy approaches. First of all, TIRFM imaging does not require extensive sample preparation as in EM studies. As the imaging is conducted in liquid (physiological buffers like PBS), the results can be more naturally attributed to physiological conditions. As the sample is still quite easily accessible while imaging, it is possible to alter the conditions (chemical environment, temperature, pH) or to add AFM for co-localized imaging. Employing co-localized TIRFM/AFM made it possible to simultaneously observe both the fluorescence signal and topography. In the recorded AFM images insulin amyloidal forms are seen as long fibers with the diameters 2–5 nm (filaments) and 10–25 nm (fibrils). These findings are in agreement with values obtained from electron microscopy studies [46,47]. By using superb sensitivity of fluorescence signal detection along with nanometer resolution, it was possible to correlate fine spectral features with topographical structure for both fibrils and filaments when coupled to the LCO probes.

In conclusion we have demonstrated a nanoscopic ultrastructural study of two distinct amyloid states formed from insulin using fluorescent probes. We envision that such a combination of biophysical techniques and amyloid specific molecular probes will be highly useful in indentifying and quantifying conformational heterogeneities among many if not all known and newly discovered amyloid proteins.

## References

[1] Chiti F., Dobson C.M. (2006). Protein misfolding, functional amyloid, and human disease. Annu. Rev. Biochem.

[2] Sipe J.D., Cohen A.S. (2000). Review: History of the amyolid fibril. J. Struct. Biol.

[3] Selkoe D.J. (1991). The molecular pathology of Alzheimer’s disease. Neuron.

[4] Westermark P. (2005). Aspects on human amyloid forms and their fibril polypeptides. FEBS J.

[5] Bucciantini M., Giannoni E., Chiti F., Baroni F., Formigli L., Zurdo J., Taddei N., Ramponi G., Dobson C.M., Stefani M. (2002). Inherent toxicity of aggregates implies a common mechanism for protein misfolding diseases. Nature.

[6] Wiréhn J., Carlsson K., Herland A., Persson E., Carlsson U., Svensson M., Hammarstrom P. (2005). Activity, folding, misfolding, and aggregation *in vitro* of the naturally occurring human tissue factor mutant R200W. Biochemistry.

[7] Dobson C.M. (1999). Protein misfolding, evolution and disease. Trends Biochem. Sci.

[8] Ross C.A., Poirier M.A. (2004). Protein aggregation and neurodegenerative disease. Nat. Med.

[9] Uversky V.N., Fink A.L. (2004). Conformational constraints for amyloid fibrillation: the importance of being unfolded. Biochim. Biophys. Acta.

[10] Sorgjerd K., Klingstedt T., Lindgren M., Kågedal K., Hammarström P. (2008). Prefibrillar transthyretin oligomers and cold stored native tetrameric transthyretin are cytotoxic in cell culture. Biochem. Biophys. Res. Commun.

[11] Gharibyan A.L., Zamotin V., Yanamandra K., Moskaleva O.S., Margulis B.A., Kostanyan I.A., Morozova-Roche L.A. (2007). Lysozyme amyloid oligomers and fibrils induce cellular death via different apoptotic/necrotic pathways. J. Mol. Biol.

[12] Bamberger M.E., Harris M.E., McDonald D.R., Husemann J., Landreth G.E. (2003). A cell surface receptor complex for fibrillar beta-amyloid mediates microglial activation. J. Neurosci.

[13] Vestergaard B., Groenning M., Roessle M., Kastrup J.S., van de Weert M., Flink J.M., Frokjaer S., Gajhede M., Svergun D.I. (2007). A helical structural nucleus is the primary elongating unit of insulin amyloid fibrils. PLoS Biol.

[14] Petkova A.T., Ishii Y., Balbach J.J., Antzutkin O.N., Leapman R.D., Delaglio F., Tycko R. (2002). A structural model for Alzheimer’s beta-amyloid fibrils based on experimental constraints from solid state NMR. Proc. Natl. Acad. Sci. USA.

[15] Antzutkin O.N., Balbach J.J., Tycko R. (2003). Site-specific identification of non-β-strand conformations in Alzheimer’s *b*-amyloid fibrils by solid-state NMR. Biophys. J.

[16] Naiki H., Higuchi K., Hosokawa M., Takeda T. (1989). Fluorometric determination of amyloid fibrils *in vitro* using the fluorescent dye, thioflavine T. Anal. Biochem.

[17] Klunk W.E., Pettergrew J.W., Abraham D.J. (1995). Quantitative evaluation of congo red binding to amyloid-like proteins with a beta-pleated sheet conformation. J. Histochem. Cytochem.

[18] Lindgren M., Sorgjerd K., Hammarstrom P. (2005). Detection and characterization of aggregates, prefibrillar amyloidogenic oligomers, and protofibrils using fluorescence spectroscopy. Biophys. J.

[19] Alizadeh-Pasdar N., Li-Chan E.C.Y. (2000). Comparison of protein surface hydrophobicity measured at various pH values using three different fluorescent probes. J. Agric. Food Chem.

[20] Wierenga P.A., Meinders M.B.J., Egmond M.R., Voragen A.G.J. (2003). Protein exposed hydrophobicity reduces the kinetic barrier for adsorption of ovalbumin to the air-water interface. Langmuir.

[21] Luk K.C., Hyde E.G., Trojanowski J.Q., Lee V.M.-Y. (2007). Sensitive fluorescence polarization technique for rapid screening of α-synuclein oligomerization/fibrillization inhibitors. Biochemistry.

[22] Orte A., Birkett N.R., Clarke R.W., Devlin G.L., Dobson C.M., Klenerman D. (2008). Direct characterization of amyloidogenic oligomers by single-molecule detection. Proc. Natl. Acad. Sci. USA.

[23] Nilsson K.P.R. (2009). Small organic probes as amyloid specific ligands—Past and recent molecular scaffolds. FEBS Lett.

[24] Nilsson K.P.R., Olsson J.D.M., Stabo-Eeg F., Lindgren M., Konradsson P., Inganas O. (2005). Chiral recognition of a synthetic peptide using enantiomeric conjugated polyelectrolytes and optical spectroscopy. Macromolecules.

[25] Åslund A., Herland A., Hammarström P., Nilsson K.P.R., Jonsson B.H., Konradsson P. (2007). Studies of luminescent conjugated polythiophene derivatives: Enhanced spectral discrimination of protein conformational states. Bioconjug. Chem.

[26] Åslund A., Nilsson K.P.R., Konradsson P. (2009). Fluorescent oligo and poly-thiophenes and their utilization for recording biological events of diverse origin—when organic chemistry meets biology. J. Chem. Biol.

[27] Sigurdson C.J., Nilsson K.P.R., Hornemann S., Manco G., Polymenidou M., Schwarz P., Leclerc M., Hammarström P., Wüthrich K., Aguzzi A. (2007). Prion strain discrimination using luminescent conjugated polymers. Nat. Methods.

[28] Sigurdson C.J., Nilsson K.P.R., Hornemann S., Heikenwalder M., Manco G., Schwarz P., Ott D., Rülicke T., Liberski P.P., Julius C. (2009). De novo generation of a transmissible spongiform encephalopathy by mouse transgenesis. Proc. Natl. Acad. Sci. USA.

[29] Nilsson K.P.R., Åslund A., Berg I., Nyström S., Konradsson P., Herland A., Inganäs O., Stabo-Eeg F., Lindgren M., Westermark G.T. (2007). Imaging distinct conformational states of amyloid-beta fibrils in Alzheimer’s disease using novel luminescent probes. ACS Chem. Biol.

[30] Nilsson K.P.R., Hammarström P., Ahlgren F., Herland A., Schnell E.A., Lindgren M., Westermark G.T., Inganäs O. (2006). Conjugated polyelectrolytes—conformation-sensitive optical probes for staining and characterization of amyloid deposits. ChemBioChem.

[31] Stabo-Eeg F., Lindgren M., Nilsson K.P.R., Inganäs O., Hammarström P. (2007). Quantum efficiency and two-photon absorption cross-section of conjugated polyelectrolytes used for protein conformation measurements with applications on amyloid structures. Chem. Phys.

[32] Åslund A., Sigurdson C.J., Klingstedt T., Grathwohl S., Bolmont T., Dickstein D.L., Glimsdal E., Prokop S., Lindgren M., Konradsson P. (2009). Novel pentameric thiophene derivatives for *in vitro* and *in vivo* optical imaging of a plethora of protein aggregates in cerebral amyloidoses. ACS Chem. Biol.

[33] Zako T., Sakono M., Hashimoto N., Ihara M., Maeda M. (2009). Bovine insulin filaments induced by reducing disulfide bonds show a different morphology, secondary structure, and cell toxicity from intact insulin amyloid fibrils. Biophys. J.

[34] Klingstedt T., Åslund A., Simon R.A., Johansson L.B.G., Mason J.J., Nyström S., Hammarström P., Nilsson K.P.R. (2011). Synthesis of a library of oligothiophenes and their utilization as fluorescent ligands for spectral assignment of protein aggregates. Org. Biomol. Chem.

[35] Kumar S., Nussinov R. (2002). Close-range electrostatic interactions in proteins. ChemBioChem.

[36] Horovitz A., Serrano L., Avron B., Bycroft M., Fersht A.R. (1990). Strength and co-operativity of contributions of surface salt bridges to protein stability. J. Mol. Biol.

[37] Yang H., Fung S.Y., Pritzker M., Chen P. (2007). Surface-assisted assembly of an ionic-complementary peptide: Controllable growth of nanofibers. J. Am. Chem. Soc.

[38] Sletmoen M., Skjåk-Bræk G., Stokke B.T. (2004). Single-molecular pair unbinding studies of mannuronan C-5 epimerase AlgE4 and its polymer substrate. Biomacromolecules.

[39] Lindgren M., Hammarström P. (2010). Amyloid oligomers: Spectroscopic characterization of amyloidogenic protein states. FEBS J.

[40] Fukuma T., Mostaert A.S., Serpell L.C., Jarvis S.P. (2008). Revealing molecular-level surface structure of amyloid fibrils in liquid by means of frequency modulation atomic force microscopy. Nanotechnology.

[41] Blackley H.K.L., Sanders G.H.W., Davies M.C., Roberts C.J., Tendler S.J.B., Wilkinson M.J. (2000). In situ atomic force microscopystudy of β-amyloid fibrillization. J. Mol. Biol.

[42] Adamcik J., Jung J.-M., Flakowski J., De Los Rios P., Dietler G., Mezzenge R. (2010). Understanding amyloid aggregation by statistical analysis of atomic force microscopy images. Nat. Nanotechnol.

[43] Sedman V.L., Adler-Abramovich L., Allen S., Gazit E., Tendler S.J.B. (2006). Direct observation of the release of phenylalanine from diphenylalanine nanotubes. J. Am. Chem. Soc.

[44] Jimenez J.L., Nettleton E.J., Bouchard M., Robinson C.V., Dobson C.M., Saibil H.R. (2002). The protofilament structure of insulin amyloid fibrils. Proc. Natl. Acad. Sci. USA.

[45] Zako T., Sakono M., Kobayashi T., Sörgerd K., Nilsson K.P.R., Hammarström P., Lindgren M., Maeda M (2012). Cell Interaction study of amyloid by using luminescent conjugated polythiophene: Implication that amyloid cytotoxicity is correlated with prolonged cellular binding. ChemBioChem.

[46] Brange J., Andersen L., Laursen E.D., Meyn G., Rasmussen E. (1997). Toward understanding insulin fibrillation. J. Pharmac. Sci.

[47] Nielsen L., Frokjaer S., Carpenter J.F., Brange J. (2001). Studies of the structure of insulin fibrils by Fourier transform infrared (FTIR) spectroscopy and electron microscopy. J. Pharmac. Sci.

